# Bayesian dynamic modeling of time series of dengue disease case counts

**DOI:** 10.1371/journal.pntd.0005696

**Published:** 2017-07-03

**Authors:** Daniel Adyro Martínez-Bello, Antonio López-Quílez, Alexander Torres-Prieto

**Affiliations:** 1 Departament d’Estadística i Investigació Operativa, Facultat de Matemàtiques, Universitat de València, València, Spain; 2 Secretaría de Salud del Departamento de Santander, Bucaramanga, Colombia; Institute for Disease Modeling, UNITED STATES

## Abstract

The aim of this study is to model the association between weekly time series of dengue case counts and meteorological variables, in a high-incidence city of Colombia, applying Bayesian hierarchical dynamic generalized linear models over the period January 2008 to August 2015. Additionally, we evaluate the model’s short-term performance for predicting dengue cases. The methodology shows dynamic Poisson log link models including constant or time-varying coefficients for the meteorological variables. Calendar effects were modeled using constant or first- or second-order random walk time-varying coefficients. The meteorological variables were modeled using constant coefficients and first-order random walk time-varying coefficients. We applied Markov Chain Monte Carlo simulations for parameter estimation, and deviance information criterion statistic (DIC) for model selection. We assessed the short-term predictive performance of the selected final model, at several time points within the study period using the mean absolute percentage error. The results showed the best model including first-order random walk time-varying coefficients for calendar trend and first-order random walk time-varying coefficients for the meteorological variables. Besides the computational challenges, interpreting the results implies a complete analysis of the time series of dengue with respect to the parameter estimates of the meteorological effects. We found small values of the mean absolute percentage errors at one or two weeks out-of-sample predictions for most prediction points, associated with low volatility periods in the dengue counts. We discuss the advantages and limitations of the dynamic Poisson models for studying the association between time series of dengue disease and meteorological variables. The key conclusion of the study is that dynamic Poisson models account for the dynamic nature of the variables involved in the modeling of time series of dengue disease, producing useful models for decision-making in public health.

## Author summary

Time series analysis of dengue disease case counts are currently employed to establish associations between dengue disease and environmental, socioeconomic and climatic variables and to predict the evolution of dengue epidemics. Nowadays there is acceptance that climatic factors like environmental temperature, rainfall and relative humidity modify the behavior of the dengue vectors, affecting the transmission of the disease. Thus, in the absence of vector data, climatic factors are commonly used to input transmission models of dengue disease on several temporal and spatial scales. We applied hierarchical Bayesian dynamic generalized models to dengue diseases case counts in a medium-sized city in Colombia, with constant and time-varying coefficients for calendar trend, and constant and time-varying coefficients for meteorological variables (temperature, rainfall, solar radiation and relative humidity). We selected a final model useful for exploring of the time-varying association between climatic variables and dengue, and the short-term out-of-sample predictions of dengue counts within the study period. We illustrate the modeling process so a data analyst on a multidisciplinary research team could integrate a time series model accounting for the time-varying nature of the data.

## Introduction

Dengue is an arboviral disease caused by a *Flavivirus*, leading to high morbidity in children and adults in tropical countries of Asia and Latin America [1]. There are four genetically distinct but antigenically related (different serotypes) dengue viruses named DEN-1, DEN-2, DEN-3, and DEN-4. All serotypes can cause a spectrum of illness ranging from unapparent or mild fever to the potentially fatal syndrome characterized by hemorrhage, fever and shock syndrome [2]. The infective female *Aedes aegypti* mosquito is the main vector involved in transmiting the viruses causing dengue. The mosquito acquires the virus when it feeds on the blood of an infected human. Several studies show that climate is associated with the mosquito ecology, the infectious agents they carry, and the arboviral transmission of dengue disease [3] [4] [5]. Naish *et al*.(2014) [3] reviewed the studies associating climatic factors and dengue transmission, concluding that higher temperatures affect the rate of larval development, shorten the emergence of adult mosquitoes, increase the biting behavior of mosquitos, and accelerates virus replication within the mosquitos. Meanwhile, the combined effect of temperature and relative humidity impact mosquito feeding behavior, vector survival and the probability to be infected and the ability to transmit dengue.

Epidemiological research on dengue incidence is based on passive surveillance data from case reports [5] [6]. Racloz *et al*. (2012) [5] reviewed early warning modelling in dengue disease, concluding that epidemiological modeling is constrained by limited data sources. Authors encouraged the collection of information at the spatial and temporal level of climatic and socio environmental variables to develop models with stronger predictive capabilities, while Runge-Ranzinger *et al*.(2014) [6] concluded that passive surveillance provides the baseline for outbreak alert, which should be strengthened through the definition of appropriate alert thresholds.

For the time series analysis of dengue case counts associated with meteorological variables, diverse methodologies have been employed, including auto-regressive integrated moving average (ARIMA) models [7] [8] [9] [10] [11] [12] [13] [14] [15], Poisson multivariate regression forecasting models [16] [17] [18], distributed lag non-linear models (DLNM) [19] [20], decision trees with cross-validation [21], multiresolution analysis and fuzzy systems [22], stepwise negative binomial multivariate linear regression analysis [23], wavelet time series analysis [24], probabilistic random walks [25] [26], and dynamic generalized linear models (DGLM) [27] [28] [29].

DGLMs are extensions of the dynamic linear models [30] [31], based on two sets of equations, a measurement or observation equation and the transition or state equations. The observation equation establishes a link between observations and unobserved variables, and the transition equations describe the evolution of state variables. DGLMs allow the inclusion of components modeling seasonality, trend, cyclicity and covariates [31]. The classic models for calendar trend are the first-order random walk model, the local linear trend model (first-order random walk plus trend) and the second-order random walk [32]. Modeling seasonality and cyclicity is accomplished through dummy variables or trigonometric series defined in the transition equations, and covariates are included with constant or time-varying coefficients [32].

DGLM parameter estimations have followed different approaches. Linear Bayes estimation with conjugate updating [30] [31] or iteratively weighted Kalman filter and smoother, accompanied by the expectation-maximization (EM) algorithm for the estimation of unknown hyperparameters [32], was applied by Chiogna and Gaetan [33] to explore the association between pollution covariates and respiratory diseases. Shepard *et al*. [34] applied likelihood base inference for non-Gaussian state space parameters, based on importance sampling.

DGLMs estimated by Markov Chain Monte Carlo (MCMC) simulations have been explored by Gamerman [35], Ferreira and Gamerman [27] (modeling Dengue disease and meningitis with covariates and seasonal terms), Schmidt and Pereira [28] and Alves *et al*. [36] including covariates with constant coefficients for time accompanied by covariates modeled by transfer functions. Malhão *et al*. [29] implemented DGLM for time series of dengue cases, capturing temporal dependencies not explained by covariates, and modeling dengue over-mortality.

Colombia is one of the countries with the highest incidence of dengue disease in the tropics, and it is testing dengue control by vaccination [37], a topic of interest among the research community [38]. The country possesses climatic, environmental and socio-geographic conditions favoring the growth and development of the dengue vector. The *Aedes aegypti* mosquito is found across more than 80% of the territory, which has an altitude of 1000 m and 2200 m above sea level, and the *Aedes albopictus* (forest and urban dengue vector) has also been reported [39].

Bucaramanga is among the Colombian cities with the highest annual dengue incidence for the 2008–2015 period. In 2010 and 2012 the city experienced incidence rates of 1515 and 279.93 cases per 100,000 people, respectively, while for the same years the incidence rates for the country were 657 and 221.9 cases per 100,000, respectively [39] [40]. The *Aedes aegypti* mosquito has been reported as the dengue vector in the city of Bucaramanga. While vectorial surveillance studies did not exist in 2008–2015 to quantify the presence of vectors, their abundance, occurrence, distribution and other epidemiological parameters at monthly or weekly temporal scales for Bucaramanga, information of climatic variables such as environmental temperature, rainfall, solar radiation, and relative humidity are available from several sources at these temporal scales. These data offer opportunities to analyze the relation between time series of dengue cases and climatic variables, as Rúa-Uribe *et al*.(2013) [8] show for another Colombian city.

The aim of this study is to model the association between time series of dengue case counts and meteorological variables, in a high-incidence city of Colombia, applying Bayesian hierarchical dynamic generalized linear models, during the period January 2008 to August 2015. Additionally, we evaluate the model’s performance in short-term prediction of dengue cases.

## Materials and methods

### Data

Bucaramanga is a medium-sized city in Colombia, at 959 meters above sea level, with a population of 527,913 people (projected population, 2015), at the coordinates 7°07′07″N, 73°06′58″W. We collected dengue case counts for 2008–2015 in metropolitan Bucaramanga from the Surveillance National System of Public Health (SIVIGILA). The total dengue case counts (probable and confirmed cases of dengue and severe dengue plus dengue mortality) by epidemiological week (EW) were computed in the interval between the first EW of January 2008 to the last EW of August 2015, for a total of 396 EW. For the meteorological variables (MV), daily maximum temperature (°C), daily total rain fall (mm), daily maximum solar radiation (Watts/m^2^) and daily maximum relative humidity (%) were obtained from three stations of the Defense Corporation of the Bucaramanga Plateau (CDMB). Daily maximum temperature (°C) and daily total rain fall (mm/m^2^) were obtained from the Institute of Hydrology, Meteorology and Environmental Studies of Colombia (IDEAM) for two meteorological stations. Daily values for every variable were averaged by EW and by station, and then the weekly averages of all stations were averaged, obtaining one value per MV and EW.

### Hierarchical dynamic Poisson models

We fitted Bayesian hierarchical dynamic Poisson models to dengue case counts. Let *y*_*t*_ be the case count for dengue in EW *t* (*t* = 1, ⋯, *T* and *T* = 396), and

$$\begin{array}{c} y_{t}∼\text{Poisson}\left(\lambda_{t}\right) \end{array}$$ (1)

The logarithm of the mean λ_*t*_ is modeled with two options. The first option is the inclusion of a constant coefficient *α* for the calendar trend,

$$\begin{array}{c} \text{log}\left(\lambda_{t}\right)=\{\begin{array}{c} \alpha \\ \alpha +\sum_{j=1}^{J}\beta_{j}x_{t-1,j}\\ \alpha +\sum_{j=1}^{J}b_{t,j}x_{t-1,j} \end{array} \end{array}$$ (2)

where *α* is Normal with mean 0 and variance 10, which allows flexibility for the exploration of the parameter space. The second option is the inclusion of time-varying coefficients *α*_*t*_ for the calendar trend,

$$\begin{array}{c} \text{log}\left(\lambda_{t}\right)=\{\begin{array}{c} \alpha_{t}\\ \alpha_{t}+\sum_{j=1}^{J}\beta_{j}x_{t-1,j}\\ \alpha_{t}+\sum_{j=1}^{J}b_{t,j}x_{t-1,j} \end{array} \end{array}$$ (3)

where the time-varying coefficients *α*_*t*_ are defined with Normal random walk 1 (RW1) or Normal random walk 2 (RW2) priors. The Normal RW1 priors for *α*_*t*_ are defined as

$$\begin{array}{c} \alpha_{1}∼\text{Normal}\left(3,0.2\right) \end{array}$$

$$\begin{array}{c} \alpha_{t}∼\text{Normal}\left(\alpha_{t-1},\tau_{\alpha }\right);\;\text{(}2\leq t\leq T\text{)} \end{array}$$

and the Normal RW2 priors for *α*_*t*_ follow

$$\begin{array}{c} \alpha_{1},\alpha_{2}∼\text{Normal}\left(3,0.2\right) \end{array}$$

$$\begin{array}{c} \alpha_{t}∼\text{Normal}\left(2\alpha_{t-1}-\alpha_{t-2},\tau_{\alpha }\right);\;\text{(}3\leq t\leq T\text{)} \end{array}$$

where for the Normal(3,0.2) prior, the mean of 3 for *α*_1_ and *α*_2_ in the exponential scale is close to the observed dengue case counts at time points 1 and 2, and 0.2 is a precision (variance of 20) that allows flexibility for these parameters. *τ*_*α*_ is the precision parameter with Gamma(1,0.1) hyperprior, which represents a Gamma prior noninformative distribution centered at 10 with variance of 100. In Eqs 2 and 3, the *x*_*t*−1,*j*_ (*j* = 1, ⋯, *J* and *J* = 4) are the mean centered MVs temperature (*j* = 1), rainfall (*j* = 2), solar radiation (*j* = 3) and relative humidity (*j* = 4). The *β*_*j*_ are constant coefficients for lag-one MV, and *b*_*t*,*j*_ are time-varying coefficients for lag-one MV. Normal priors with mean 0 and variance 10 were assigned to the constant coefficients *β* for the covariates. The time-varying coefficients for the lag-one covariates received first-order Normal RW1 priors,

$$\begin{array}{c} b_{1,j}∼\text{Normal}\left(0,0.1\right) \end{array}$$

$$\begin{array}{c} b_{t,j}∼\text{Normal}\left(b_{t-1,j},\tau_{b_{j}}\right)\;\text{(}2\leq t\leq T\text{)} \end{array}$$

where for the Normal(0,0.1), we let *b*_1,*j*_ start centered at zero, with a 0.1 precision (variance of 10), allowing a large space for exploring the parameter. Gamma(1,0.001) prior distributions (Gamma centered at 1000 with variance of 100,000) are assigned to the precision parameters *τ*_*b*_*j*__. The reason for this prior is that we constrain the variance of the *b*_*t*,*j*_ to be very small, smoothing the trend of the time-varying coefficients and allowing us to visualize the smoothed trend of the covariate effects.

We modeled missing data in the covariates by imputing the empty values, assuming a Normal(*μ*_*t*−1_, *τ*_*j*_) prior for *t* = 1, ⋯, *T* and *T* = 396, where *μ*_*t*−1_ is the value of the lag-one week meteorological centered variable, where *τ*_*j*_ is a precision parameter with Gamma(0.1,0.1) priors for temperature, for rainfall, solar radiation, and relative humidity, where the Gamma prior is an informative prior centered at 0.1 with dispersion 10, slightly constraining the imputed values of the covariates to have a small variance, without restricting to high variance values.

Models were fitted applying MCMC using WinBUGS 1.4 software [41], with 3 chains, 50,000 iterations total, 46,000 iterations burn-in and thinning of 4, obtaining a final sample of 1000 iterations per chain. Convergence was assessed by Gelman-Rubin diagnostic [42] and visual inspection of the simulations chains. Model selection was accomplished using deviance information criteria (DIC) [43]. When DIC measures are used for model selection, models with small deviance $\bar{\text{D}}$, a small number of parameters *p*_*D*_ and a small DIC are selected for inference.

After fitting all models, and selecting the final model for inferences, we were interested in evaluating the short-term prediction performance of the selected final model.

We obtained predictions at several time points, during the study period *T* = 396. We selected estimation periods 1 to *t*, where *t* was in increments of 20 EWs, starting in the 20th EW of the study period and ending in the 380th EW. We obtained 19 upper bounds for the estimation period 1 to *t*.

Then we fitted models for periods 1 to *p*, where *p* = *t* + *k* (*k* = 1, ⋯, 4), and the *k* are prediction periods (one, two, three or four weeks ahead). We used the same conditions defined above for the MCMC simulations. Samples from the posterior predicted distribution for the prediction periods *k* were obtained, and the mean and 95% credible intervals (CIs) for the cases of dengue were calculated. To evaluate the prediction performance from the final model, we calculated the mean absolute percentage error (MAPE) per MCMC iteration between the predicted cases of dengue *y*_*pred*_*k*__ and the observed case count *y*_*k*_, at prediction periods *k* (∑_*k*_ |(*y*_*pred*_*k*__ − *y*_*k*_)/*y*_*k*_|/*k*). We present the median MAPE of the posterior predictive distribution for all the estimation periods *t* for one, two, three and four weeks ahead as a measure of short-term model performance for predicting dengue case counts.

## Results

### Exploratory data analysis

**Fig 1:**
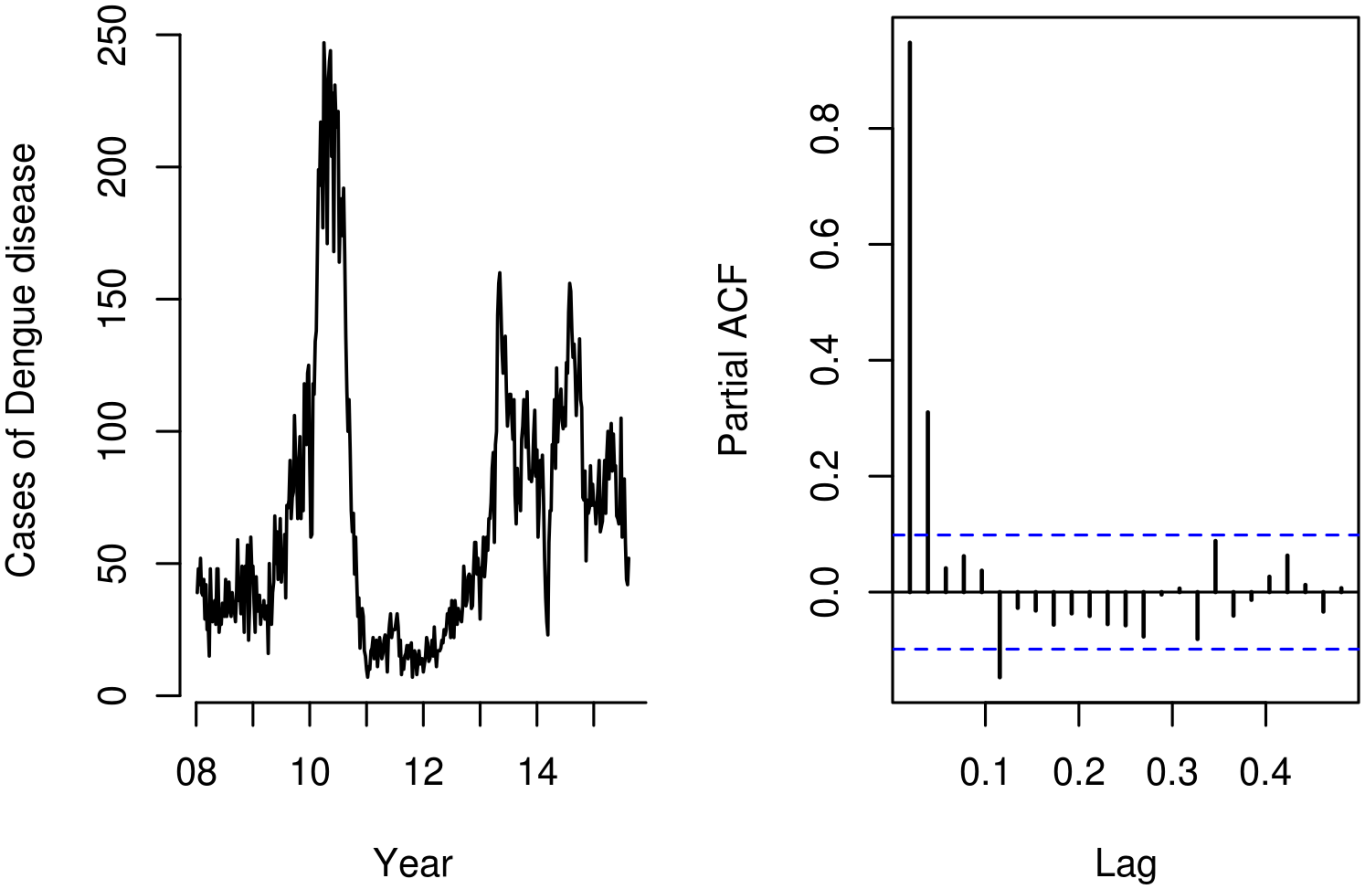
Dengue time series plots. Time series plot of dengue case counts (left) and partial autocorrelation function plot of dengue case counts (right).

The total number of cases of dengue disease for the study period was 26,755. The weekly case count averaged 67.6, with a median of 52 (range 7 to 247). There were three dengue disease outbreaks in 2010, 2013 and 2014, with small case counts in 2011 and 2012 (Fig 1). The partial autocorrelation function for the time series of dengue case counts (Fig 1) suggest a first- or second-order autoregressive process.

Maximum weekly temperature averaged 27°C, with a minimum of 23.6°C, a maximum of 30.4°C, and 18 missing values. Mean and median values of weekly rainfall were 2.7 mm/m^2^ and 3.6 mm/m^2^, respectively, with a minimum of 0, a maximum of 24.8 mm/m^2^, and 11 missing values. Weekly maximum solar radiation averaged 946.5 Watts/m^2^, with median of 940.9 Watts/m^2^, a minimum of 733.5 Watts/m^2^, a maximum of 1279 Watts/m^2^, and 66 missing values. Maximum weekly relative humidity averaged 94.2%, with a minimum of 79.2%, a maximum of 99.5%, and 63 missing values.

**Fig 2:**
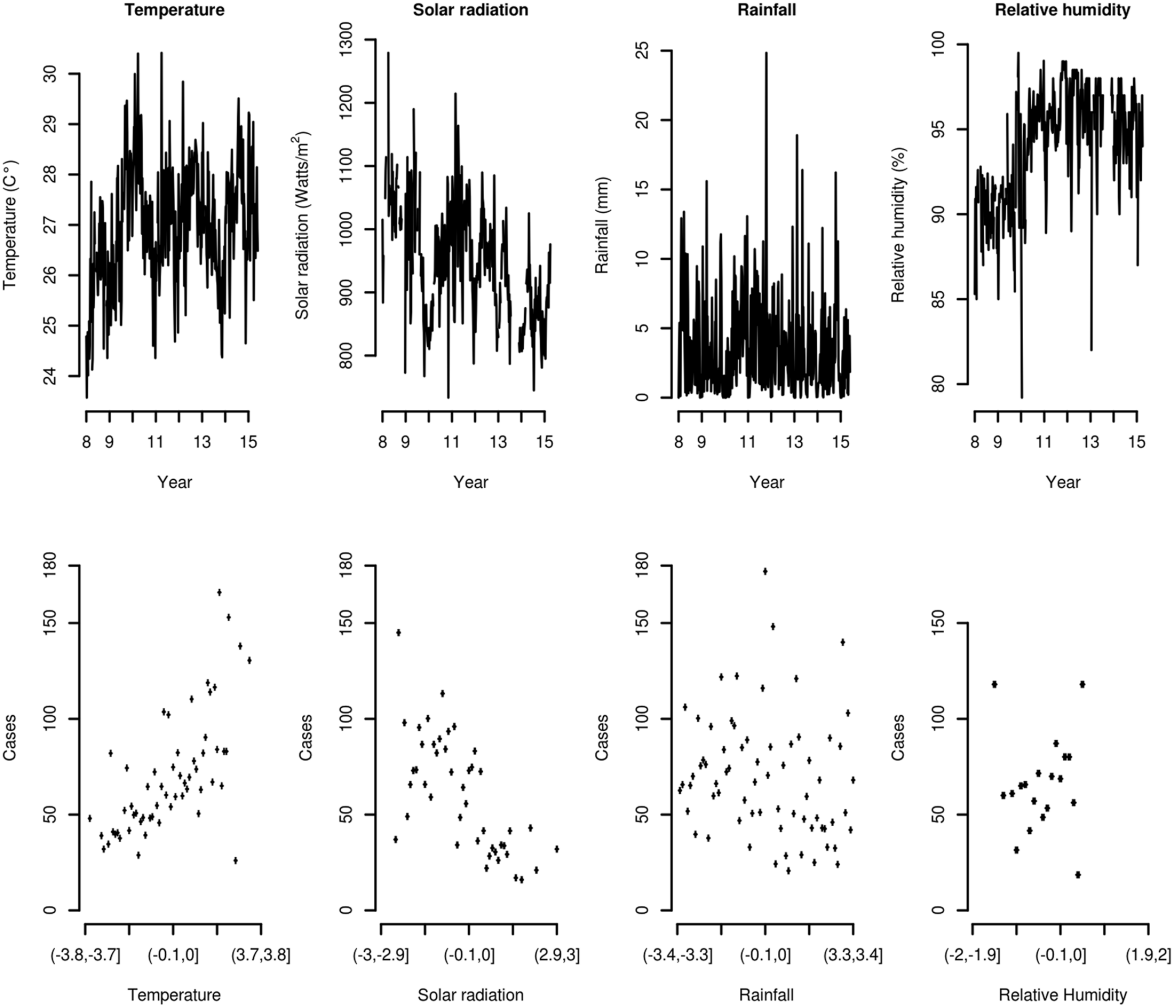
Meteorological variables time series plots. Time series plots of temperature, rainfall, solar radiation and relative humidity (top) and scatter plots of the average number of cases of dengue by intervals of the meteorological variables (bottom)

Fig 2 shows plots of time series for MVs, and plots of the average dengue case counts by intervals of the MVs.

While time series for temperature and relative humidity display an upward trend over the 396 EWs, solar radiation decreases, and precipitation shows highly volatile behavior. Dengue disease case counts are positively correlated with temperature, and negatively correlated with solar radiation. There is no apparent association between dengue case counts and precipitation or relative humidity.

**Fig 3:**
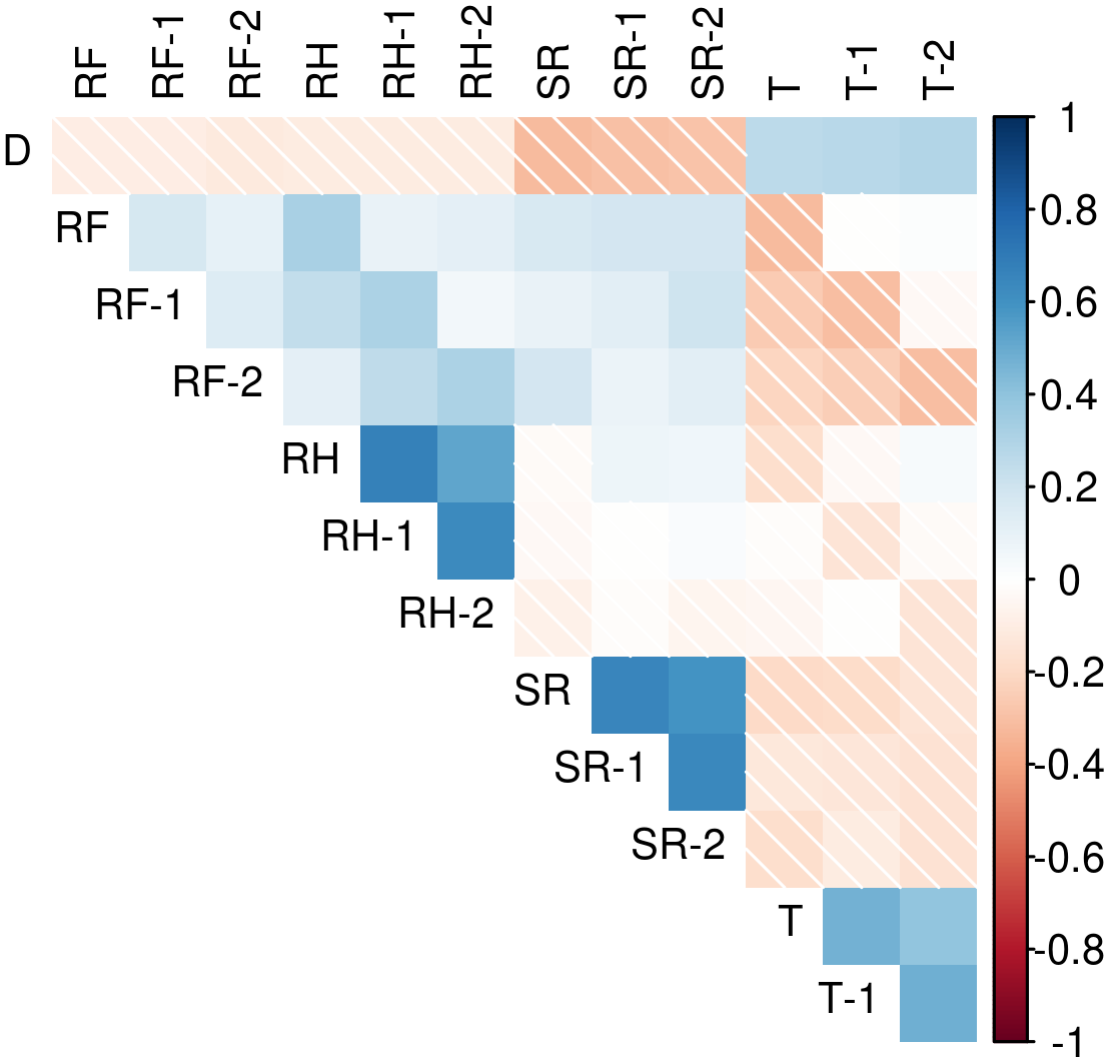
Correlation matrix plot of weekly dengue case counts and lag-zero, lag-one and lag-two meteorological variables. D: dengue disease cases. RF: rainfall. RH: relative humidity. SR: solar radiation. T: temperature.

In Fig 3, linear correlations between the meteorological variables and dengue case counts show positive and moderate correlation with temperature and negative and moderate linear correlation with relative humidity, solar radiation and rainfall. Relative humidity and solar radiation display high positive correlations with their own lag-1 and lag-2 values, followed by temperature and rainfall. Rainfall, relative humidity and solar radiation are positively and moderately correlated, while rainfall and temperature show negative and moderate correlation. Finally, we highlight the negative and low correlation between solar radiation and temperature.

### Dynamic Poisson models

In this section, we begin by presenting the results from the models without covariates (only constant coefficient (CC) (*α*) or RW1 or RW2 time-varying coefficients (TVCs) (*α*_*t*_) for calendar trend). We define calendar trend as the pattern observed in the model’s parameters over the EWs in the entire study period (2008–2015), not the trends observed over any given epidemiological year. We then present the results from models including CC (*β*_*j*_) for covariates, and CC (*α*) or RW1 or RW2 TVCs (*α*_*t*_) for calendar trend. Finally, we exhibit the results from models including RW1 TVCs (*b*_*t*,*j*_) for the covariates with CC (*α*) or RW1 or RW2 TVCs (*α*_*t*_) for calendar trend.

#### Models without covariates

For the models without covariates, the deviance and DIC for the model with CC (*α*) for calendar trend are 15,959.8 and 15,960.8, respectively. For the models with RW1 or RW2 TVCs (*α*_*t*_) for trend, the respective deviance and DIC are 2716.4 and 2901.1 for the RW1 model, and 2901.5 and 2990.0 for the RW2 model. We conclude that the model with CC (*α*) for trend shows worse fit than the models with RW1 or RW2 TVCs (*α*) for trend of calendar time. The models with RW1 or RW2 TVCs (*α*_*t*_) for calendar trend have similar DIC, while the model with RW1 TVCs (*α*_*t*_) for calendar trend offers the best fit (small deviance).

#### Models with CC (*β*_*j*_) for the covariates

Table 1 presents the DIC selection measures from the simple (single covariate) Poisson regression models with CC (*β*_*j*_) for the covariates, and CC (*α*) or RW1 or RW2 TVCs (*α*_*t*_) for calendar trend.

**Table 1:** DIC measures for models with constant coefficient (*α*), RW1 or RW2 TVCs (*α*_*t*_) for calendar trend with CC (*β*_*j*_) for the covariates.

	*β*_Temperature_			*β*_Rainfall_			*β*_Solar radiation_			*β*_Relative humidity_		
Trend	$\bar{\text{D}}$	*p*_*D*_	DIC	$\bar{\text{D}}$	*p*_*D*_	DIC	$\bar{\text{D}}$	*p*_*D*_	DIC	$\bar{\text{D}}$	*p*_*D*_	DIC
*α*	14341.9	12.5	14354.4	15735.2	6.3	15741.4	13627.1	46.3	13673.4	15260.3	-590.4	14669.9
*α*_*t*_ (RW1)	2713.4	188.9	2902.3	2717.4	185.2	2902.6	2717.4	184.9	2902.3	2717.7	184.9	2902.7
*α*_*t*_ (RW2)	2836.2	119.5	2955.8	2908.3	88.7	2997.0	2905.6	89.5	2995.1	2898.9	90.8	2989.8

First, for every meteorological variable, the model with CC (*α*) for calendar trend and CC (*β*_*j*_) for the covariates corresponds to the simple Poisson regression, while the models with RW1 or RW2 TVCs (*α*_*t*_) for trend and CC (*β*_*j*_) for the covariates are the simple dynamic Poisson regression.

Second, the simple Poisson regression models display worse fit than the simple dynamic Poisson regression models, evidenced by high DIC and deviance values.

Third, the fit of the simple Dynamic Poisson models with CC (*β*_*j*_) for the covariates, and RW1 TVCs (*α*_*t*_) for calendar trend is better than models with RW2 TVCs (*α*_*t*_) for calendar trend.

Table 2 displays parameter estimates of the CC (*β*_*j*_) for the covariates, from models with CC (*α*) or RW1 or RW2 TVCs (*α*_*t*_) for calendar trend, from Table 1. Parameter estimates for the CC for temperature are 0.207 (95% CI: 0.197, 0.217); solar radiation, -0.309 (95% CI: -0.324, -0.294); and rainfall -0.026 (95% CI: -0.030, -0.022), from models with CC (*α*) for calendar trend suggesting a strong association between these variables and the weekly case counts of dengue.

**Table 2:** Parameter estimates of models with CC (*α*) or RW1 or RW2 TVCs (*α*_*t*_) for calendar trend and CC (*β*_*j*_) for the covariates.

Trend	Mean	SD	95% CI	Trend	Mean	SD	95% CI
	*β*_Temperature_				*β*_Solar radiation_		
*α*	0.207	0.005	(0.197, 0.217)	*α*	-0.309	0.007	(-0.324, -0.294)
*α*_*t*_ (RW1)	0.010	0.013	(-0.014, 0.035)	*α*_*t*_ (RW1)	-0.001	0.022	(-0.046, 0.040)
*α*_*t*_ (RW2)	0.006	0.011	(-0.017, 0.027)	*α*_*t*_ (RW2)	-0.010	0.022	(-0.047, 0.035)
	*β*_Rainfall_				*β*_Relative humidity_		
*α*	-0.026	0.002	(-0.030, -0.022)	*α*	0.009	0.026	(-0.029, 0.031)
*α*_*t*_ (RW1)	0.001	0.003	(-0.005, 0.007)	*α*_*t*_ (RW1)	-0.003	0.005	(-0.012, 0.007)
*α*_*t*_ (RW2)	0.001	0.002	(-0.004, 0.006)	*α*_*t*_ (RW2)	-0.007	0.004	(-0.015, 0.000)

There is no statistical association between cases of dengue disease and relative humidity (0.026, 95% CI: -0.029, 0.031). These parameters correspond to the simple Poisson regression model.

Although models with CC (*α*) for calendar trend show strong statistical association between covariates and dengue, the point estimates and 95% CIs from models with RW1 or RW2 TVCs (*α*_*t*_) for trend show a weak association between cases of dengue and the meteorological variables, while these models present the best fit (small DIC and deviance).

#### Models with RW1 TVCs (*b*_*t*,*j*_) for the covariates

Next, we fitted models with CC (*α*) or RW1 or RW2 TVCs (*α*_*t*_) for calendar trend, with RW1 TVCs (*b*_*t*,*j*_) for the lag-one covariates. Information criteria for these simple dynamic Poisson regression models with TVCs (*b*_*t*,*j*_) for the covariates are presented in Table 3. For temperature, DIC for the models with CC (*α*) or RW2 TVCs (*α*_*t*_) for calendar trend are higher than the model with RW1 TVCs (*α*_*t*_) for calendar trend.

**Table 3:** DIC measures for models with CC (*α*) or RW1 or RW2 TVCs (*α*_*t*_) for calendar trend with RW1 TVCs (*b*_*t*,*j*_) for the covariates.

	*b*_*t*,Temperature_			*b*_*t*,Rainfall_			*b*_*t*,Solar radiation_			*b*_*t*,Relative humidity_		
Trend	$\bar{\text{D}}$	*p*_*D*_	DIC	$\bar{\text{D}}$	*p*_*D*_	DIC	$\bar{\text{D}}$	*p*_*D*_	DIC	$\bar{\text{D}}$	*p*_*D*_	DIC
*α*	2872.0	314.8	3186.8	2989.4	322.0	3311.5	3177.6	38.8	3216.4	3030.0	-539.1	2490.9
*α*_*t*_ (RW1)	2710.2	187.7	2897.9	2706.7	189.4	2896.1	2709.7	172.4	2882.1	2705.7	176.9	2882.5
*α*_*t*_ (RW2)	2841.3	103.5	2944.8	2846.2	114.3	2960.5	2783.8	84.6	2868.4	2807.6	94.2	2901.8

DIC for rain fall display similar results as temperature, i.e., DIC for the model with CC (*α*) or RW2 TVCs (*α*_*t*_) for trend are higher than the model with RW1 TVCs (*α*_*t*_) for calendar trend.

For solar radiation, DIC for the model with RW2 TVCs (*α*_*t*_) for calendar trend is smaller than the models with RW1 TVCs (*α*_*t*_) and CC (*α*) for calendar trend.

Lastly, the model with RW1 TVCs for relative humidity plus CC (*α*) for calendar trend have the smallest DIC for this covariate (DIC = 2490.9), but the number of parameters (*p*_*D*_) is negative (*p*_*D*_ = -539.1), which makes this model a poor option. DIC from the models with RW1 or RW2 TVCs (*α*_*t*_) for calendar trend do not present negative *p*_*D*_. The smallest DIC is for the model with RW1 TVCs (*α*_*t*_) for calendar trend.

**Fig 4:**
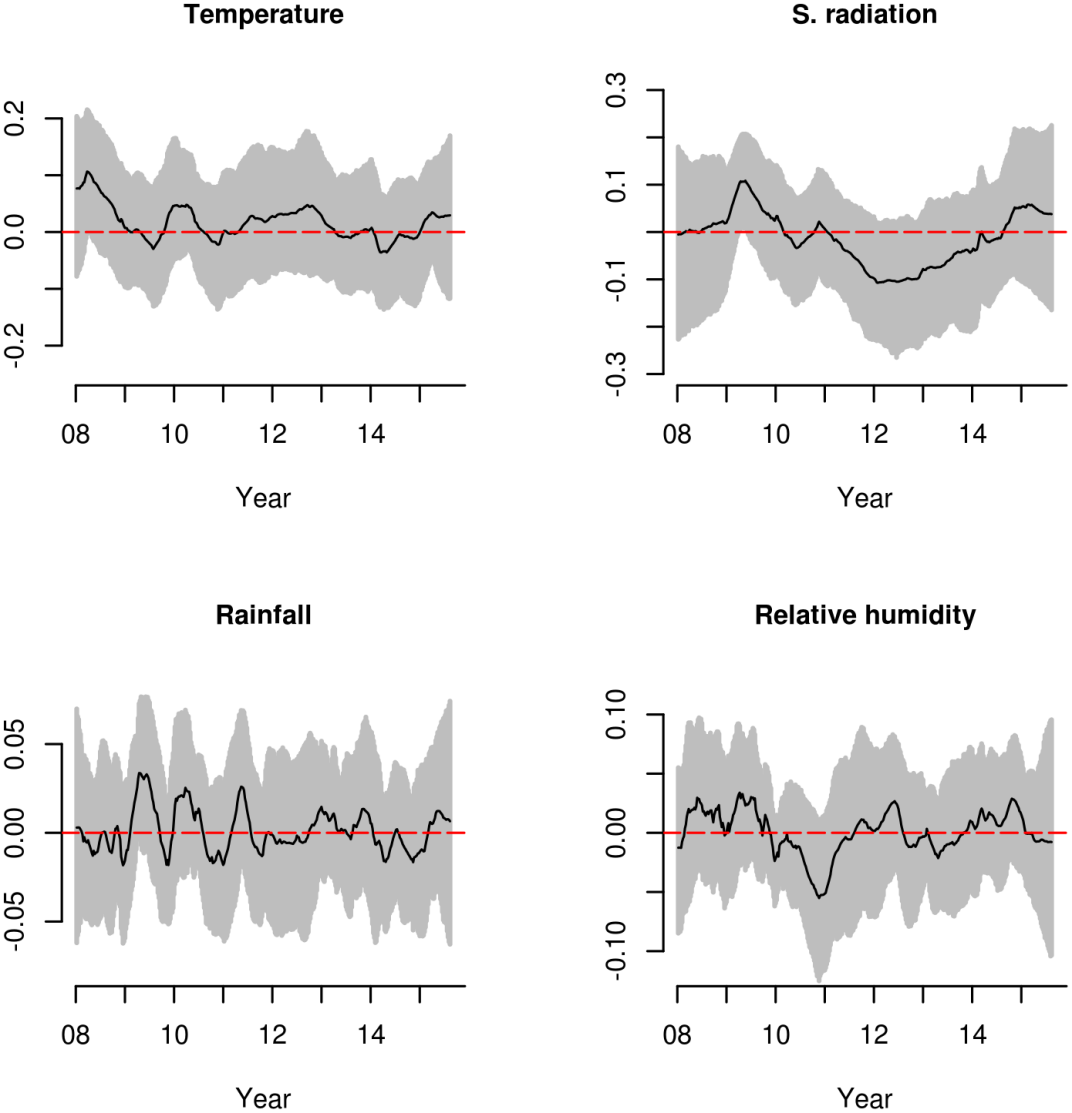
Mean and 95% CI for the TVCs (*b*_*t*,*j*_) for temperature, rainfall, solar radiation and relative humidity from the saturated model.

At this stage of the analysis, we identified models with RW1 TVCs (*α*_*t*_) for calendar trend plus RW1 TVCs (*b*_*t*,*j*_) for the covariates, as the models offering the best fit (smallest deviance and DIC). Then, in addition to the simple dynamic Poisson regression models with TVCs (*b*_*t*,*j*_) for the covariates, we fitted multiple (multiple variables) dynamic Poisson models, presenting the information criteria in Table 4. DIC measures for all the models with RW1 TVCs (*α*_*t*_) for trend plus RW1 TVCs (*b*_*t*,*j*_) for the meteorological variables range from 2831.4 to 2897.6 (Table 3). The model with RW1 TVCs for solar radiation and relative humidity (*b*_*t*,*SR*_ + *b*_*t*,*RH*_) presents the smallest DIC (DIC = 2831.4) and effective number of parameters (*p*_*D*_ = 133.5), followed by the model including all the MVs in the predictors (*b*_*t*,*T*_ + *b*_*t*,*RF*_ + *b*_*t*,*SR*_ + *b*_*t*,*RH*_) (DIC = 2847.2), which presents the smallest deviance, selecting this saturated model for inference instead of model with solar radiation and relative humidity, because the model with the lowest DIC is also the model with the most imputed variables (solar radiation and relative humidity). We include the WinBUGS code for the selected model in S1 File, and convergence diagnostic measures in S1 Appendix for the model parameters in Table 4. Finally, from the model with TVCs for all the meteorological variables (*b*_*t*,*T*_ + *b*_*t*,*RF*_ + *b*_*t*,*SR*_ + *b*_*t*,*RH*_) in Table 3, we plot the time-varying parameter estimates (mean and 95% CIs) in Fig 4.

**Table 4:** DIC selection measures from models with RW1 TVCs (*α*_*t*_) for calendar trend and RW1 TVCs (*b*_*t*,*j*_) for the covariates. *b*_*t*,*T*_: temperature. *b*_*t*,*RF*_: rainfall. *b*_*t*,*SR*_: solar radiation. *b*_*t*,*RH*_: relative humidity.

Model	$\bar{\text{D}}$	*p*_*D*_	DIC
*b*_*t*,*T*_	2710.2	187.7	2897.9
*b*_*t*,*RF*_	2706.7	189.4	2896.1
*b*_*t*,*SR*_	2709.7	172.4	2882.1
*b*_*t*,*RH*_	2705.7	176.9	2882.5
*b*_*t*,*T*_ + *b*_*t*,*RF*_	2699.7	196.0	2895.7
*b*_*t*,*T*_ + *b*_*t*,*SR*_	2701.8	183.3	2885.1
*b*_*t*,*T*_ + *b*_*t*,*RH*_	2699.8	179.4	2879.2
*b*_*t*,*P*_ + *b*_*t*,*SR*_	2696.9	182.8	2879.7
*b*_*t*,*RF*_ + *b*_*t*,*RH*_	2694.8	182.8	2877.6
*b*_*t*,*SR*_ + *b*_*t*,*RH*_	2697.9	133.5	2831.4
*b*_*t*,*T*_ + *b*_*t*,*RF*_ + *b*_*t*,*SR*_	2687.2	191.3	2878.5
*b*_*t*,*T*_ + *b*_*t*,*RF*_ + *b*_*t*,*RH*_	2686.9	192.5	2879.4
*b*_*t*,*RF*_ + *b*_*t*,*SR*_ + *b*_*t*,*RH*_	2684.8	182.7	2867.6
*b*_*t*,*T*_ + *b*_*t*,*SR*_ + *b*_*t*,*RH*_	2692.8	178.9	2871.7
*b*_*t*,*T*_ + *b*_*t*,*RF*_ + *b*_*t*,*SR*_ + *b*_*t*,*RH*_	2680.9	166.3	2847.2

TVCs for temperature and solar radiation present higher variability than the coefficients for relative humidity and rainfall. Point estimates for temperature start at values higher than zero, in contrast with relative humidity, solar radiation and rainfall, which begin almost at zero. TVCs for temperature are above zero for 2008 and 2010, below zero for 2009 and 2014, and close to zero for 2011 to 2013 and for the year 2015, with 95% CIs not including zero only for 2008.

TVCs for solar radiation are above zero for 2009 and 2015, with a small peak in 2010, and below zero for 2011 to 2014, with the 95% CIs including zero for the entire study period, with the exception of 2009.

For rainfall, TVCs present high volatility, with coefficients above zero for 2009, 2010, 2011, 2014 and 2015, and below zero for 2008, 2012 and 2014, with 95% CIs including zero for all years in the study period except 2009.

TVCs for relative humidity are above zero for 2008, 2009 and 2012 and below zero for 2010, 2011 and 2013; the 95% CIs cross zero for the complete study period.

#### Short-term prediction of dengue case counts

**Fig 5:**
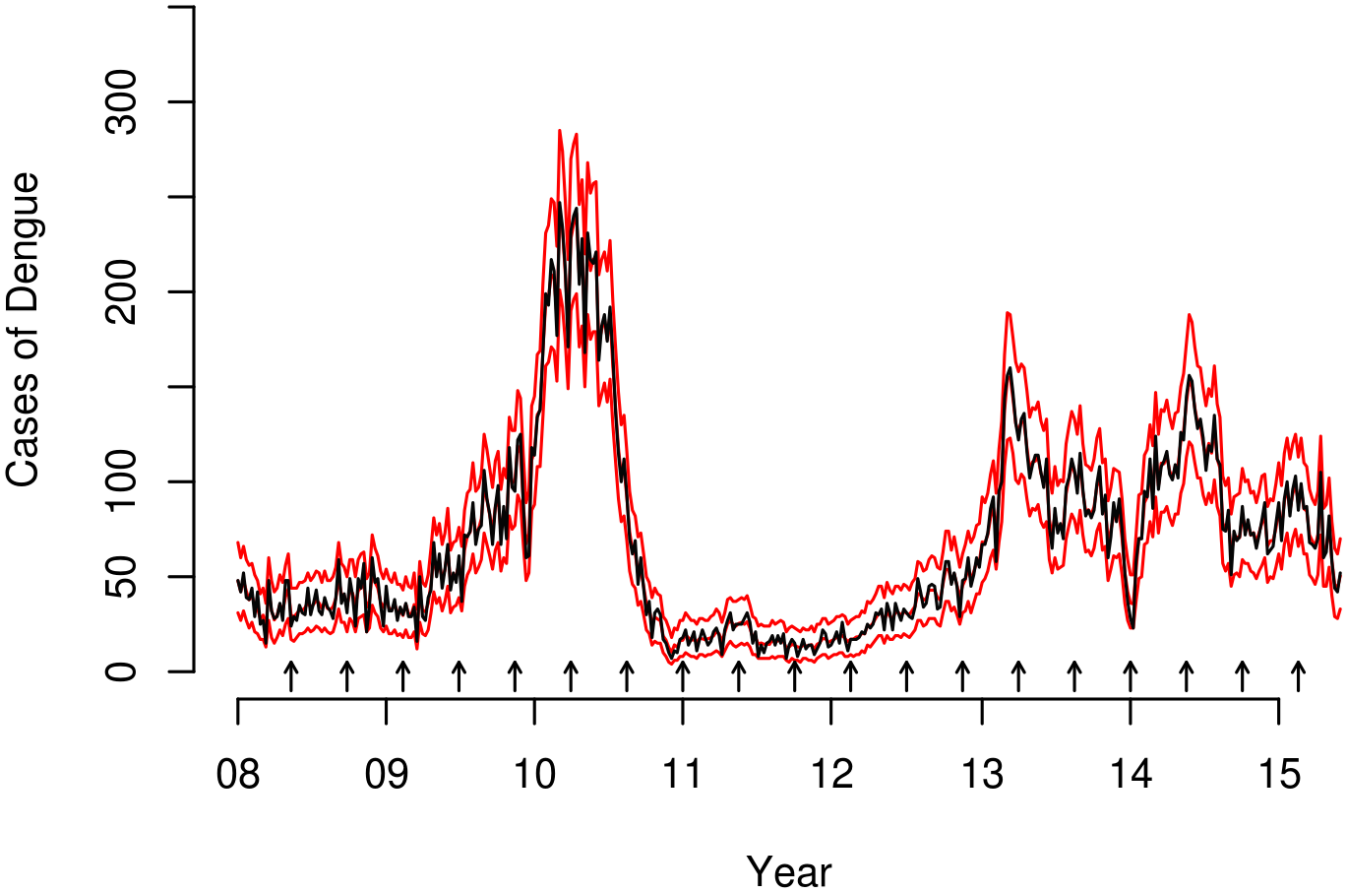
Mean and 95% CI for the predicted case counts of dengue disease (red lines) from the selected model, and observed counts (gray line). Arrows representing the EW were short-term predictions of dengue case counts at one, two, three and four weeks.

We use the model with RW1 TVCs (*α*_*t*_) for calendar trend plus TVCs (*b*_*t*,*j*_) for the covariates ($\text{log}\left(\lambda_{t}\right)=\alpha_{t}+\sum_{j=1}^{4}b_{t,j})$ (*j* = 1, temperature; *j* = 2, rainfall; *j* = 3, solar radiation; *j* = 4, relative humidity) to obtain a forecast for several time points during the study period 1 to *T* (*T* = 396). Fig 5 presents the observed and predicted dengue case counts obtained for the selected final model. Based on Fig 5, we can distinguish the trend of the dengue case counts in the time periods close to the prediction points: from June to December 2008, the trend was stable. Then, there was a gradual increase in May 2009 and a sharp rise in November 2009. Afterwards, the trend stabilized, but then became highly volatile in May 2010 (at the peak of the 2010 outbreak) before slowly decreasing in November 2010. Between May and October 2011, the trend was stable, showing a slow increase from April to October 2012. The trend from March to September 2013 is a rapid decrease, followed by a rapid increase in March 2014, and a slow decrease in September 2014, before evening out in March 2015.

**Fig 6:**
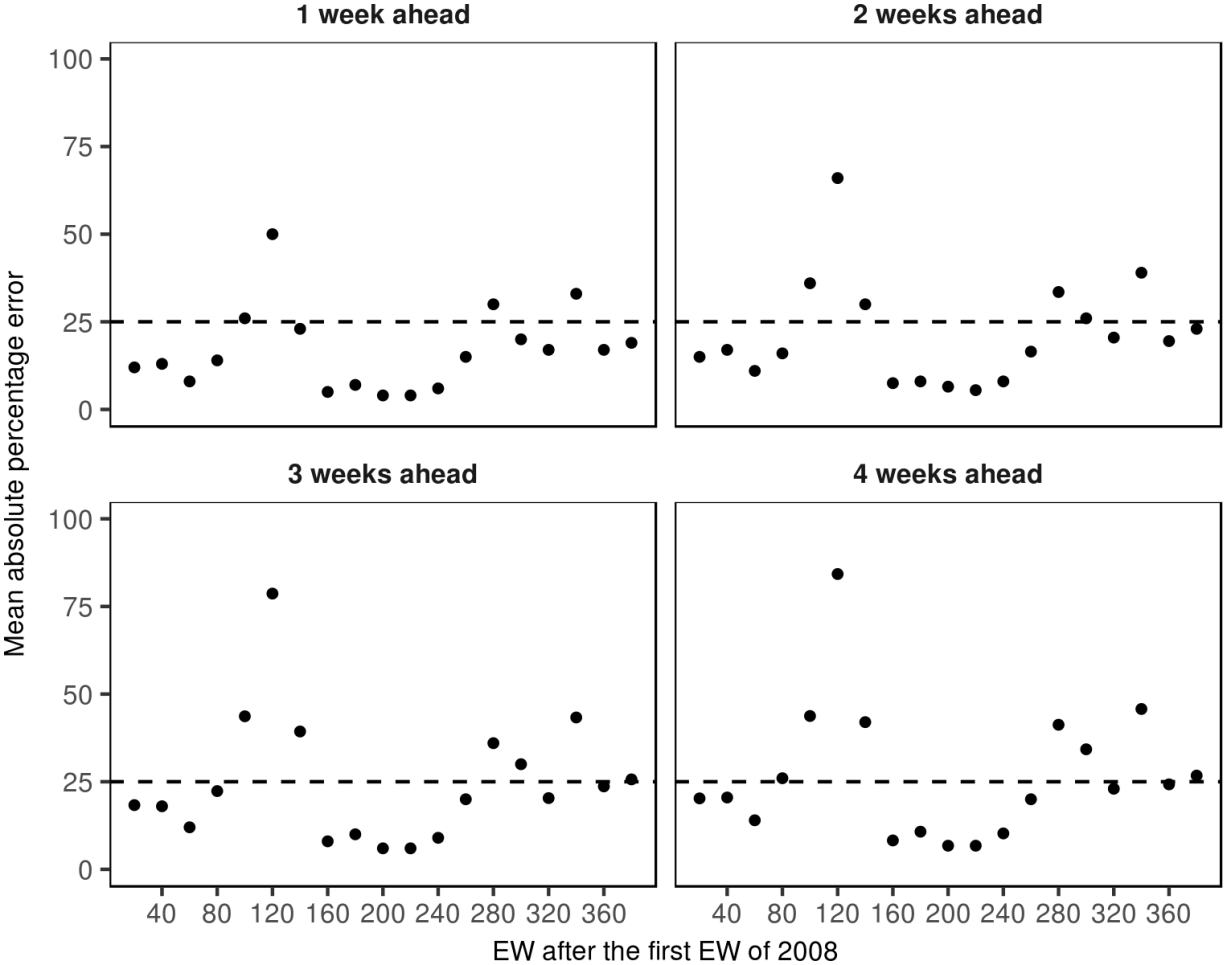
Median of the MCMC simulations for the mean absolute percentage error (MAPE) to evaluate the short-term predictive performance of the final model in selected EWs after the first EW of January 2008.

Table 5 presents the MAPE between the predicted mean and the observed dengue case counts for short-term prediction periods at one, two, three and four weeks, estimated at selected EW after the first EW of 2008, from the model selected for inferences. A quick inspection reveals that the highest MAPEs correspond to the EW associated with outbreaks in 2010, 2013 and 2014. Fig 6 show the MAPE results presented in Table 5.

**Table 5:** Median of the MCMC simulations for the mean absolute percentage error (MAPE) to evaluate the short-term predictive performance of the final model in selected EWs after the first EW of January 2008.

			Weeks ahead			
Year	Date	EW after first EW 2008	1	2	3	4
2008	May 11	20	12.0	15.0	18.3	20.2
	Sep 28	40	13.0	17.0	18.0	20.5
2009	Feb 15	60	8.0	11.0	12.0	14.0
	Jul 05	80	14.0	16.0	22.3	26.0
	Nov 22	100	26.0	36.0	43.7	43.8
2010	Apr 11	120	50.0	66.0	78.7	84.2
	Aug 29	140	23.0	30.0	39.3	42.0
2011	Jan 01	160	5.0	7.5	8.0	8.2
	Jun 06	180	7.0	8.0	10.0	10.8
	Oct 10	200	4.0	6.5	6.0	6.8
2012	Mar 03	220	4.0	5.5	6.0	6.8
	Jul 07	240	6.0	8.0	9.0	10.2
	Dec 12	260	15.0	16.5	20.0	20.0
2013	Jun 05	280	30.0	33.5	36.0	41.2
	Sep 09	300	20.0	26.0	30.0	34.2
2014	Feb 02	320	17.0	20.5	20.3	23.0
	Jun 06	340	33.0	39.0	43.3	45.8
	Nov 11	360	17.0	19.5	23.7	24.2
2015	Apr 04	380	19.0	23.0	25.7	26.8

In the Figure, we added an horizontal line at 25% to help the inspection of the MAPEs. We conclude that for most periods, the MAPEs are under 25%, meaning that if we fitted the model for different estimation periods over the course of the study (January 2008 to August 2015) we could estimate the observed dengue case count for one or two weeks ahead with an error no more than 25%.

## Discussion

In this report, DGLMs are employed to model time series of dengue disease case counts and meteorological variables. DGLMs for the data at hand included two components: the first substracts the temporal pattern, and the second models the covariate effect. We observed weak time-varying associations between cases of dengue disease and solar radiation and temperature. Time-varying associations mean that the dengue case counts are associated with solar radiation and temperature changes over time, where some intervals show a positive association, while in other intervals the association is negative. DGLMs are a straightforward way to deal with count data, without the need to transform or alter the response variable, accounting for covariates with natural time-varying behavior.

For parameter estimation, we applied MCMC using WinBUGS 1.4, providing the flexibility to include constant and time-varying coefficients for calendar trend and covariates. There are few examples of studies including time-varying coefficients. Lee and Shaddick (2008) [44] fit DGLMs to pollution data and respiratory diseases, based on the block sampling algorithm from Knorr-Held (1999) [45]. Ruiz-Cardenas *et al*. (2012) [46] employed Integrated Laplace Approximation (INLA) to illustrate the fit of simulated and real time series of counts, using augmented data with the inclusion of time varying-coefficients for calendar trend and covariates.

Our findings can be summarized as follows: in the models without covariates, the best model was the RW1 TVCs (*α*) for trend. Within the models with CC (*β*_*j*_) for covariates, we found the worst fit in models with CC (*α*) for trend, which display strong association (95% CIs not including zero) between weekly cases of dengue and temperature, solar radiation and rainfall, but not with relative humidity. However, models with RW1 or RW2 TVCs (*α*_*t*_) for calendar trend had a good fit, revealing a weak association between dengue and the covariates. These findings are important because simple and multiple Poisson regression models with constant coefficients for the covariates are statistical methods commonly employed to model counts of infectious diseases like dengue [4].

For example, Hii *et al*. [16] modeled dengue and weather variables, applying a Poisson multiple regression model with piecewise linear spline functions for the covariates and constant coefficient terms to model autoregression, seasonality and trend. They validated the model by forecasting cases of dengue for week 1 of 2011 up to week 16 of 2012 using weather data alone.

In the class of models with RW1 TVCs (*b*_*t*,*j*_) for the covariates, the best model corresponds to the simple dynamic Poisson model with RW1 TVCs (*α*_*t*_) for calendar trend. After fitting the simple dynamic regression models, we fitted multiple dynamic regression models, with several combinations of TVCs (*b*_*t*,*j*_) for the covariates, and we selected the model including all the meteorological variables. Our final model delineates the time-varying association between the covariates and cases of dengue, although the inspection of the mean estimates and 95% CIs of the RW1 TVCs (*b*_*t*,*j*_) for the covariates shows a weak association.

In the literature associating dengue and weather variables, many of the modeling strategies show strong association (evidenced by low p-values) between dengue and meteorological variables, with different lag periods. As an example, Xu *et al*. [19] established an association between absolute humidity (relative humidity adjusted by temperature) and dengue cases using a Poisson distributed lag non-linear model, with cubic splines for the covariates and accounting autoregression with constant coefficients for the lag-one and lag-two response.

We also evaluate the short-term predictive performance of the selected model, concluding that it enables relatively accurate (< 25% error) prediction of weekly dengue case counts at one or two weeks ahead although the predictions are strongly influenced by volatility in the weeks preceding the prediction periods, with high volatility associated with high MAPE in the predictions, as occurred in the peak of the 2010, 2013 and 2014 outbreaks in Bucaramanga.

Before finishing our discussion, we acknowledge some study limitations. The dengue case counts used in the data corresponded to the probable and confirmed cases reported to the official public health surveillance system in Colombia. The weekly dengue data was the sum of the the dengue and severe dengue cases per EW. Romero-Vega *et al*. (2014) [47] concluded that the expansion factor (the factor by which the reported cases should be multiplied to adjust for underreporting) of dengue was 7.6 for 2013, which is high. This implies that efforts to decrease underreporting must be undertaken to improve data quality for the entire surveillance system. It would be difficult to quantify the impact of underreporting in our conclusions, but still, the methods we used are valid for adjusted time series of dengue.

The covariates data (time series of temperature, rainfall, solar radiation and temperature) were a composition of several time series at daily and hourly temporal scales from several meteorological stations at different locations in the city. We summarized the data, averaging them for the different temporal scales and stations and consequently losing some data. However at some point the analyst must decide how to summarize the information to input variables for a modeling exercise. If the temporal scale is reduced (from weekly to daily data) the dengue case counts will be lower, and the Poisson models presented in the study could fit the data much better than Normal models.

One of this study’s referees remarked on the absence of vector data in the study. We explored several sources of vector data in the city, but we did not find any data at the temporal scale of the study. We recognize that the inclusion of data for the distribution, presence and ecology of the vector would improve the conclusions of the study, but this is an opportunity to show that dengue in Colombia, and particularly in Bucaramanga, is a neglected disease, despite its huge impact on the population and the allocation of resources for dengue research (Villabona-Arenas *et al*., 2016) [38].

One interesting experience in ongoing vectorial surveillance is in the city of Medellín, Colombia. Rúa-Uribe (2016) [48] reported that the Health Office of this city designed an entomological surveillance system using mosquito larval traps. We hope that the results of this interaction between the public sector and the research community will be disseminated to the country, and similar surveillance systems will be applied in all Colombian cities affected by arboviral diseases.

In the mean-time, for the city of Bucaramanga, we applied a dynamic Poisson model with time-varying coefficients for the covariates and calendar trend, which helps to establish the association between climatic factors and dengue case counts at a small temporal scale, providing a prediction model within the bounds of the limitations presented in the study.

Forecasting models are commonly deployed in dengue research literature. Earnest *et al*. [10] compare the forecasting ability of the ARIMA model and the two-component Knorr-Held model (seasonal and epidemic Bayesian hierarchical time series model) to predict out-of sample cases of dengue. They found similar predictive ability (lower MAPE values) for the Bayesian K-H model and the ARIMA model.

Forecasting models of dengue disease usually account cyclical or seasonal behavior of the time series at hand. Earnest *et al*. [10] and Hii *et al*. [16] included seasonal trend by means of sinusoidal terms with trigonometric series structure. In a previous stage, we included seasonal terms, but we removed them from the models, allowing the time-varying coefficients for calendar trend alone account for dengue incidence trends. We establish the short-term predictive performance of a model with time-varying coefficients (*α*_*t*_) for calendar trend and time-varying coefficients (*b*_*t*,*j*_) for meteorological covariates. We found a moderate predictive ability from the model to forecast cases of dengue disease at one or two weeks, which could be used by public health authorities interested in employing predictive models to help in the labors of dengue surveillance and control in Colombia.

For the future, we will explore the study models in different datasets from other cities of Colombia because, the enviromental and physical conditions are generally similar between many cities and municipalities. The models presented in the study are not only available for use with climatic variables. They can also include data from vectorial studies, socioeconomic variables and many more, if these are available at weekly or monthly temporal scales. In conclusion, we found that dynamic generalized linear models can forecast dengue cases at one or two weeks in Bucaramanga, based on temperature, rainfall, solar radiation and relative humidity, and the models allow us to explore the association between weekly cases of dengue and these covariates through the time.

## Supporting information

S1 FileWinBUGS code.We include the .odc file containing WinBUGS code for the selected model with RW1 time-varying coefficients (*α*_*t*_) for calendar trend and RW1 time-varying coefficients (*b*_*t*,*j*_) for covariates ($\text{log}\left(\lambda_{t}\right)=\alpha_{t}+\sum_{j=1}^{4}b_{t,j})$ (*j* = 1, temperature; *j* = 2, rainfall; *j* = 3, solar radiation; *j* = 4, relative humidity).(ODC)

S1 Appendix Diagnostic measures for the model parameters convergence. (PDF)
