# Supplementary material for: Bayesian dynamic modeling of time series of dengue disease case counts
Source: PLoS Negl Trop Dis. 2017 Jul 3;11(7):e0005696. doi: 10.1371/journal.pntd.0005696 (PMC5510904; doi:10.1371/journal.pntd.0005696)
Supplement: S1 Appendix — (PDF) [file pntd.0005696.s002.pdf]

S1 Appendix
Bayesian Dynamic Modeling of Time Series of Dengue Disease Case Counts

Diagnostic measures for the model parameters convergence

We employed the Gelman-Rubin (GR) diagnostic [1] to evaluate MCMC convergence by analyzing the difference between multiple Markov chains. Values substantially above 1 indicate lack of convergence. We show in Table S1 the GR statistics for the standard deviations (σ_α, σ_T, σ_{RF}, σ_{SR} and σ_{RH}) of models with RW1 time-varying coefficients α_t for calendar trend and RW1 time-varying coefficients for the covariates. For most models, the convergence of the selected parameters are close to the value of 1, while some of the GR statistics for σ_T and σ_{SR} display values slightly above 1. We accept those values, because the standard deviations for these covariates reflect the convergence of 395 parameters each, showing that few of the time-varying coefficients present difficulties to converge, but in general we accept them. Fig S1 shows the trace plots and densities of the standard deviations (σ_α, σ_T, σ_{RF}, σ_{SR} and σ_{RH}) from the model including all the covariates $b_{t,T}+b_{t,RF}+b_{t,SR}+b_{vRH}$. We observed more volatility in the trace-plots for σ_T and σ_{SR} than for σ_α, σ_{RF}, and σ_{RH}, with densities slightly skewed to the right.

Table S1: Gelman-Rubin diagnostic for the models with RW1 time-varying coefficients α_t for calendar trend and RW1 time-varying coefficients for the covariates

Model	σ_α	σ_T	σ_{RF}	σ_{SR}	σ_{RH}
$b_{t,T}$	1.00	1.19	-	-	-
$b_{t,RF}$	1.00	-	1.03	-	-
$b_{t,SR}$	1.00	-	-	1.02	-
$b_{t,RH}$	1.00	-	-	-	1.08
$b_{t,T} + b_{t,RF}$	1.00	1.03	1.03	-	-
$b_{t,T} + b_{t,SR}$	1.00	1.08	-	1.02	-
$b_{t,T} + b_{t,RH}$	1.00	1.32	-	-	1.03
$b_{t,RF} + b_{t,SR}$	1.01	-	1.02	1.14	-
$b_{t,RF} + b_{t,RH}$	1.00	-	1.01	-	1.00
$b_{t,SR} + b_{t,RH}$	1.00	-	-	1.03	1.01
$b_{t,T} + b_{t,RF} + b_{t,SR}$	1.00	1.03	1.01	1.18	-
$b_{t,T} + b_{t,RF} + b_{t,RH}$	1.00	1.04	1.00	-	1.04
$b_{t,T} + b_{t,SR} + b_{t,RH}$	1.01	1.10	-	1.02	1.02
$b_{t,RF} + b_{t,SR} + b_{t,RH}$	1.00	-	1.00	1.16	1.02
$b_{t,T} + b_{t,RF} + b_{t,SR} + b_{t,RH}$	1.01	1.04	1.00	1.29	1.01

[1] Gelman, A and Rubin, DB. Inference from iterative simulation using multiple sequences, Statistical Science 1992; 7, 457-511.

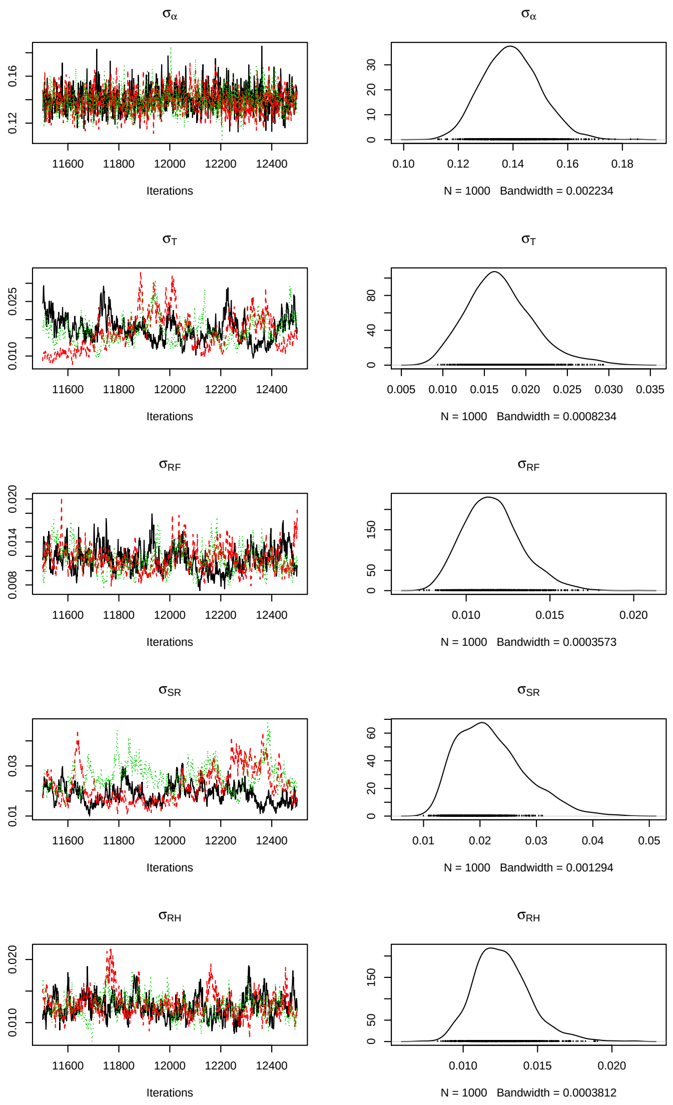

Figure S1: Trace plots and density plots for the standard deviations (σ_α, σ_T, σ_{RF}, σ_{SR} and σ_{RH}) of the selected model for inferences
